# Escharotics and Coagulants

**Published:** 1889-09

**Authors:** A. W. Harlan

**Affiliations:** Chicago.


ARTICLE V.

### ESCHAROTICS AND COAGULANTS.

READ BY DR. A. W. HARLAN, CHICAGO.

Escharotic—A substance which produces an eschar. A caustic. (Webster)—Caustic, destructive to the texture of anything; burning, corrosive, pungent, to burn. Applied to animal substances it burns, corrodes or destroys the texture, an escharotic. "Escharotics, called also cauteranis, are medicines which destroy the structure and vitality of the parts to which they are applied. The eschar which their application produces is followed by inflammation and suppuration of the surrounding tissues, by which the slough is separated from the living parts. They are employed—1. To effect the destruction of morbid growths, warts, condylomata, polypi, fungous granulations, etc.—2. To destroy the virus of rabid and venomous animals, and of chancres and malignant pustules, and to prevent their absorption. 3.—For the cure of violent inflammation, by their substitutive action, as when they are applied to the mucous or cutaneous surfaces, in gonorrhœal ophthamia, erysipelas, poisoned parts, carbuncles, etc. 4. To stimulate indolent sinuses, ulcers, etc., where this influence is also of a substitutive character. 5. To form issues. 6. To remove morbid heterologous growths, as lupus, cancer, warts, etc. (Biddle). An escharotic applied to a morbid growth or the healthy tissue incinerates it, and renders it unfit for further use. The eschar will be thrown off—it cannot be converted into living tissue by digestion or absorption. It is useless to expect it to be reconverted into pabulum. It is a foreign body which cannot be encysted and remain harmless. It is waste. It can never again serve a useful purpose. To apply an escharotic in the sense in which it is here understood and defined, is to forever destroy the part to which it is applied. When an escharotic is applied to a living pulp or the mucous, cutaneous or peridental membrane, the part is destroyed, it is not converted into anything that will perform the function of the destroyed part. This ought to be thoroughly understood.

Classification of caustics (Farquarson).

| *Penetrating Caustics.* | *Circumscribed Caustics.* |
|---|---|
| Acetic Acid. | Dried Alum. |
| Bromine. | Dried Zinc Sulphate. |
| Chromic Acid. | Heat. |
| Mercuric Nitrate Solution. | Potassa with Lime (Vienna Paste.) |
| Potassa. | Silver Nitrate. |
| Sulphuric Type-Acids. | Zinc Chloride. |

To which may be added:

| | |
|---|---|
| Caustic Soda and Lime (London Paste). | Sodium Ethylate. |
| | Carbolate of Potash. |
| Arsenic Acid. | Aseptol. |
| Phosphoric Acid. | Nitrate of Zinc. |
| Bichromate of Potassa. | Nitro-Muriatic Acid. |
| Arsenious Oxide. | Electric Cautery. |
| Cold. | |

To the above list, which is long enough, no more additions of a useful character need be made, as the remedies cannot become readily available for instant use. "Acids unite with albumen in different proportions, producing acid albumins. When mixed with blood they not only precipitate substances, but decompose hæmoglobin, forming a substance which holds oxygen with more tenacity than hæmoglobin. They coagulate myosin and produce instantaneous rigidity in muscles. Sulphuric and phosphoric acids have, besides this chemical affinity, a strong attraction for water, and completely decompose the tissues to which they are applied, so that they are powerful escharotics. Nitric acid does not readily redissolve the albumem precipitated by it, and thus forms a barrier to its own action, so that it does not penetrate so deeply as sulphuric acid."

(Brunton). In Dental Surgery the use of escharotics for their destructive effects is not very common, most practitioners relying on the knife for the purpose of removing small or large tumors and other excrescences. I do not intend to dwell on the general use of escharotics at great length, but rather devote my time to pointing out the abuses of escharotics in minor dental surgery. The most common abuse of escharotics may be found to consist in their application to exposed pulps and the peridental membrane. In this connection it may be well to remark that this is not the form of abuse of escharotics which needs the severest condemnation, but it is in repeated application of such powerful medicaments in full strength, or only slightly diluted, that the greatest danger lies. Their application to such finely organized structures as the dental pulp and the peridental membrane, unless for purposes of total destruction, I deem to be unwise and contrary to our knowledge of the properties of escharotics. Escharotics in full strength should never be used on the normal dental pulp, the mucous or peridental membrane. Escharotics are not indicated in pulpless teeth. A pulpless tooth may serve as a channel through which escharotics may be pumped or syringed into diseased tracts beyond the apices of roots, but if you wish to bleach a tooth well, do not use an escharotic in contact with the dentine, as it will prevent the diffusion of bleaching agents, until the charred organic matter is first mechanically removed. Powerful escharotics disorganize blood and blood serum, pus and living tissue as well, whether it be normal or pathological, hence the necessity for caution in their application to an already dead pulp, the bleeding end of the stump of a pulp, a serous exudate or pus flowing from an abscess beyond the apex of a root. Escharotics should be used to destroy exuberant growths, for stimulation of sluggish parts, and to prevent the absorption of pus or septic matter.

*Coagulants.*—substances which produce coagulation. To cause to change into a curd-like or inspissated state, to thicken, concrete, curdle. (Webster). All alcoholic tinctures are coagulants. All fluid extracts containing alcohol are coagulants. Nearly all escharotics, even in very weak aqueous solution, are coagulants. Some other substances (astringents) may coagulate albumen or blood serum to a degree unexpected by the users, as I shall endeavor to show at the conclusion of this paper. Rubefacients and vesicants are not always coagulants. Example, essential oils, chloroform, ether, etc.

Coagulation is sometimes produced by a feeble oxidation as by the air. In other cases by the formation of albuminous pellicles, as may be seen after the application of tincture of iodine or dilute solution of silver nitrate. Carbolic acid dilute, five per cent. or 95 per cent. produces a carbolate of albumen; this is a chemical change. Other coagulants act by their chemical affinities on the tissues to which they are applied, as by the abstraction of water, thereby concreting the substance acted upon. Coagulation does not always prevent the coagulum from being converted into new tissue, as may be noted in the healing of arteries and other well substantiated cases. The coagulum may be absorbed without elimination entire. Coagulation is a necessary condition before some articles of food can be digested. Some coagula may be converted into a lower order of tissue which will serve the purposes of protection, as witness the successful capping of pulps. A coagulum is however an effectual barrier to the immediate penetration of medicaments in roots of teeth for the purpose of disinfection, and should not be produced. It may become a source of future trouble by disorganizing in the presence of anærobic microbes.

Coagulation of blood serum or a serous exudate is one of the factors in pulp preservation. A successful treatment of many lesions of the mucous membrane and peridental membrane, depends on the degree of coagulation produced by the application of coagulants. Coagulation is defined to be protective, not destructive. Violent escharotics, tissue destroyers, if used at all as coagulators, must be largely diluted. Many of the true escharotics are not freely soluble in water, hence the difficulty of properly diluting them to serve as useful coagulators. A pulp accidentally uncovered in excavating, but not bleeding, does not need a coagulator to protect it from the air, as the air is a coagulant in a minor degree. Hence the 1-1000 bichloride solution may be used without danger to its external covering. When the pulp is exposed and bleeding, first arrest the hæmorrhage by the use of a true astringent, then apply a feeble coagulating stimulant and cover without pressure. When suppuration is found, the neutral peroxide of hydrogen is indicated, which should be followed with a combination of non-irritant dressings, as Dr. Black's 1-2-3, solution of iodoform in ether or iodoform with essential oils. Carbolic acid, 95 per cent., is not indicated in a case of this kind. Aqueous solution of beta-naphthol or hydro-naphthol may be used. Wood creosote or guaiacol, likewise. A 10 per cent. solution of resorcin or pheno-resorcin may be used with good results. Mucous abrasions, erosions and "canker" sore mouth need to be touched with glycerole of thymol, full strength, resorcin, acid aromatic sulph., 1-2-3; solution of permanganate of potash, 1-500 corrosive sublimate or similar stimulating coagulants. That degree of coagulation of the surfaces of pockets around the roots of teeth which is best, is attained by the direct application of medicaments with a syringe or the introduction of finely-pointed medicated gelatine bougies, as recommended by Dr. W. B. Ames. Coagulation in cases of this kind, must not be destructive, When possible, the coagulation should be accomplished by the use of substances soluble in water or alcohol—preferably in water. If the coagulant is dissolved in an acid it may cause trouble by rendering the cementum sensitive, and redissolving the coagulum before the acid is dissipated by the admixture with saliva. Coagulation should follow the cleansing of surfaces, not precede it. The coagulum should not be disturbed, nature will take care of that. Coagulators should not be applied to the pulp of a tooth every day, nor on the mucous membrane. It is not good practice to inject coagulators into pyorrhœa pockets oftener than once in four days. A coagulator should possess the following properties: Stimulating, alterative, anæsthetic, disinfectant and tonic when applied to assist in a return of the tissue to a normal condition. Coagulators should not be burners of tissue. Coagulators of albumen must be kept out of pulpless teeth. No necessity exists for their use in a pulpless tooth. Coagulation always prevents diffusion of vapors and vaporizable substances. Non-coagulators are indicated in this class of cases. Coagulators may be used in teeth containing living pulps as in many cases they are good pain obtundents. A coagulator sufficiently powerful to curdle egg-albumen is potent for the arrest of pain in a living tooth. Powerful coagulators should not be injected into deep sinuses or abscesses having no external outlet, because of the pain produced and the danger from inflammation of subjacent parts. Such cases need to be washed or irrigated with neutral waters or very feeble coagulators in the beginning and these washings may be frequently repeated when no acute inflammation exists. The antrum of Highmore is a cavity into which it would be hazardous to inject a considerable quantity of a 10 per cent. solution of chloride of zinc, or a 20 per cent. solution of acid aromaticum sulphuricum. Weak solutions of coagulators are indicated in chronic disease of the mucous lining of the antrum, until a decided effect is sought to be produced by medication, when double or triple strength of the medicament is indicated. Syphilitic sores, when painful, must be cauterized or their surfaces coagulated, concreted. I will now perform a series of experiments on flesh with escharotics. The tissue used is from the calf. I will next show the action of coagulants on blood serum and egg albumen. These experiments will be followed with an exhibition of the action of non-coagulants on blood serum and egg albumen:

### EXPERIMENTS—*Escharotics.*

| | | |
|---|---|---|
| Chloride of Zinc | (Liquefied) | On egg-albumen. |
| Chromic Acid | " | " " |
| Caustic Potash | (Full Strength) | On Veal. |
| Arsenic Acid | " " | " |
| London Paste | " " | " |
| Vienna Paste | " " | " |
| Phosphoric Acid | " " | " |
| Bichromate Potash | " " | " |
| Acid Nitrate Mercury | " " | " |
| Silver Nitrate | " " | " |
| Nitro-Muriatic Acid | " " | " |
| Sodium Ethylate | " " | " |
| Caustic Soda | " " | " |
| Glacial Acetic Acid | " " | " |
| Bromine | " " | " |

### *Coagulants.*

| | | |
|---|---|---|
| Peroxide Hydrogen | (Aqueous Solution) | Egg-Albumen. |
| " " | " " | Blood-Serum. |
| Corrosive Sublimate | (Solution 1-500) | " " |
| " " | " " | Egg-Albumen. |
| Alcohol, | (Absolute) | " " |
| Carbolic Acid | (95 per cent.) | " " |
| " " | " " | Blood-Serum. |
| Iodide Zinc, | (Liquefied) | " " |
| " " | " | Egg-Albumen. |
| Campho-Phenique, | (Full Strength) | " " |
| Black's 1-2-3, | " " | " " |
| Carbolized Potash, | " " | " " |
| " " | " " | Blood-Serum. |
| Tincture Iodine, | " " | " " |
| " " | " " | Egg-Albumen. |
| " " Comp, | " " | " " |
| " Benzoin, " | " " | " " |
| " Myrrh, | " " | " " |
| " Opium, | " " | " " |
| " Chloride of Iron, | " " | " " |
| Dialysed " | " " | " " |

| | | |
|---|---|---|
| Aluminium Chloride, | (Liquefied) | Egg-Albumen. |
| Aromatic Sulph. Acid | (Full Strength) | " " |
| " " " | " " | Blood-Setum. |
| Sulphate Copper | (Liquefied) | Egg-Albumen. |
| Sulphate Zinc | " 1 to 8 | " " |
| Creolin | (Full Strength) | " " |
| Creosote | (Morson's Wood) | " " |
| Guaiacol | (Full Strength) | " " |
| Spirits Camphor | " " | " " |
| Tannic Acid | (Aqueous Solution) | " " |
| Gallic Acid | " " | " " |
| Hydro-Naphthol | " " | " " |
| Beta " | " " | " " |
| Menthol | (Alcoholic Solution) | " " |
| Oil of Cade | (Full Strength) | " " |
| Resorcin | (10 per cent) | " " |
| Vinum Opii | (Full Strength) | " " |
| Listerine | " " | " " |
| Soziodol | (Aqueous Solution) | " " |
| Cologne Water | (Full Strength) | " " |
| Oleo-Resin Kava-Kava | " " | " " |
| Salicylic Acid | (Aqueous Solution) | " " |
| Lactic " | " " | " " |

*Non-Coagulants.*

| | | |
|---|---|---|
| Oil Cloves | (Full Strength) | Egg-Albumen. |
| Oil Cinnamon | " " | " " |
| Oil Cassia | " " | " " |
| Oil Turpentine | " " | " " |
| Oil Camphor | " " | " " |
| Oil Cajeput | " " | " " |
| Oil Sassafras | " " | " " |
| Oil Gaultheria | " " | " " |
| Oil Sanitas | " " | " " |
| Terebene | " " | " " |
| Carbol-Camphor | " " | " " |
| Iodoform and Ether | (Saturated Solution) | " " |
| Boracic Acid | (Aqueous " | " " |

| | | |
|---|---|---|
| Chloroform | (Full Strength) | Egg-Albumen. |
| Labarraques Solution | " " | " " |
| Liquid Vaseline | " " | " " |
| Sodium Fluo-Silicate | (Aqueous Solution) | " " |
| Permanganate Potash | " " 1 to 8 | " " |
| Eugenol | (Full Strength) | " " |
| Eucalyptol | " " | " " |
| Myrtol | " " | " " |
| Benzol | " " | " " |
| Toluol | " " | " " |
| Glycerole Thymol | " " | " " |
| Boro-Glyceride | " " | " " |
| Phenol Sodique (Wildman) | " " | " " |

**NOTE.—Chloroform after it has been in contact with egg-albumen for some hours appears to be a feeble coagulator, one specimen of oil of cloves behaved in a similar manner. Eugenol after it has been exposed to the air for some months will produce a filmal coagulation, very slight it is true, but sufficient to rank it as a feeble coagulator.**

—*Proceedings of the Ill. State Den. Society.*

---

ARTICLE VI.

